# Robust ${\bf{P}}{\bf{T}}$ symmetry of two-dimensional fundamental and vortex solitons supported by spatially modulated nonlinearity

**DOI:** 10.1038/s41598-019-40752-x

**Published:** 2019-03-14

**Authors:** Eitam Luz, Vitaly Lutsky, Er’el Granot, Boris A. Malomed

**Affiliations:** 1 Department of Physical Electronics, School of Electrical Engineering, Faculty of Engineering, Tel Aviv University, Tel Aviv, 69978 Israel; 2 Department of Electrical and Electronic Engineering, Ariel University, Ariel, Israel; 3 Center for Light-Matter Interaction, Tel Aviv University, Tel Aviv, 69978 Israel

## Abstract

The real spectrum of bound states produced by ${\bf{P}}{\bf{T}}$-symmetric Hamiltonians usually suffers breakup at a critical value of the strength of gain-loss terms, i.e., imaginary part of the complex potential. The breakup essentially impedes the use of ${\bf{P}}{\bf{T}}$-symmetric systems for various applications. On the other hand, it is known that the ${\bf{P}}{\bf{T}}$ symmetry can be made unbreakable in a one-dimensional (1D) model with self-defocusing nonlinearity whose strength grows fast enough from the center to periphery. The model is nonlinearizable, i.e., it does not have a linear spectrum, while the (unbreakable) ${\bf{P}}{\bf{T}}$ symmetry in it is defined by spectra of continuous families of nonlinear self-trapped states (solitons). Here we report results for a 2D nonlinearizable model whose ${\bf{P}}{\bf{T}}$ symmetry remains unbroken for arbitrarily large values of the gain-loss coefficient. Further, we introduce an extended 2D model with the imaginary part of potential ~*xy* in the Cartesian coordinates. The latter model is not a ${\bf{P}}{\bf{T}}$-symmetric one, but it also supports continuous families of self-trapped states, thus suggesting an extension of the concept of the ${\bf{P}}{\bf{T}}$ symmetry. For both models, universal analytical forms are found for nonlinearizable tails of the 2D modes, and full exact solutions are produced for particular solitons, including ones with the unbreakable ${\bf{P}}{\bf{T}}$ symmetry, while generic soliton families are found in a numerical form. The ${\bf{P}}{\bf{T}}$-symmetric system gives rise to generic families of stable single- and double-peak 2D solitons (including higher-order radial states of the single-peak solitons), as well as families of stable vortex solitons with *m* = 1, 2, and 3. In the model with imaginary potential ~*xy*, families of single- and multi-peak solitons and vortices are stable if the imaginary potential is subject to spatial confinement. In an elliptically deformed version of the latter model, an exact solution is found for vortex solitons with *m* = 1.

## Introduction

While wave functions of quantum systems may be complex, spectra of their energy eigenvalues must be real, which is usually secured by restricting the underlying Hamiltonian to be Hermitian^1^. However, the condition of the reality of the energy spectrum does not necessarily imply that it is generated by an Hermitian Hamiltonian. Indeed, it is well known that non-Hermitian Hamiltonians obeying the parity-time (${\mathscr{P}}{\mathscr{T}}$) symmetry may also produce entirely real spectra^2–7^. In terms of the single-particle complex potential,

1 $$P({\bf{r}})\equiv V({\bf{r}})+iW({\bf{r}}),$$

the ${\mathscr{P}}{\mathscr{T}}$ symmetry requires its real and imaginary parts to be even and odd functions of coordinates^2^: $V({\bf{r}})=V(\,-\,{\bf{r}})$, $W(\,-\,{\bf{r}})=-\,W({\bf{r}})$, i.e.,

2 $$P(\,-\,{\bf{r}})={P}^{\ast }({\bf{r}}),$$

where the asterisk stands for the complex conjugate. Actually, Hamiltonians which keep ${\mathscr{P}}{\mathscr{T}}$ symmetry may be transformed into Hermitian ones^8–10^.

In the general case, the energy spectrum generated by the ${\mathscr{P}}{\mathscr{T}}$-symmetric potential remains real (physically relevant) below a certain critical value of the strength of the imaginary part of the underlying potential, *W*(**r**) in Eq. (1), which is a threshold of the ${\mathscr{P}}{\mathscr{T}}$ symmetry breaking. Above the critical value, the system is made unstable by emerging imaginary parts of energy eigenvalues. In some models, the breakup of the ${\mathscr{P}}{\mathscr{T}}$ symmetry may follow the onset of the jamming anomaly, which means a transition from increase to decrease of the power flux between the spatially separated gain and loss spots with the growth of the gain-loss coefficient^11^. The fragility of the ${\mathscr{P}}{\mathscr{T}}$ symmetry essentially limits the use of this property in applications, where new effects, such as unidirectional transmissivity^12^, enhanced absorption of light^13^, lasing in microrings^14^, acoustic sensors^15^, as well as the operation of ${\mathscr{P}}{\mathscr{T}}$-symmetric metamaterials^16^ and microcavities^17^ strengthen with the increase of the gain-loss coefficient.

Thus far, the ${\mathscr{P}}{\mathscr{T}}$ symmetry was not experimentally realized in quantum systems, and, moreover, it was argued that, strictly speaking, ${\mathscr{P}}{\mathscr{T}}$-symmetric systems do not exist in the framework of the quantum field theory^18^. On the other hand, a possibility to implement the concept of the ${\mathscr{P}}{\mathscr{T}}$ symmetry in terms of classical physics was predicted for optical media with symmetrically placed gain and loss elements^19–34^, which is based on the similarity between the Schrödinger equation in quantum mechanics and the paraxial-propagation equation for optical waveguides. Experimentally, this possibility was implemented in several waveguiding settings^35–38^, as well as in other photonic media, including exciton-polariton condensates^39,40^, and in optomechanical systems^41^. In these contexts, breaking of the ${\mathscr{P}}{\mathscr{T}}$ symmetry was observed experimentally too. Emulation of the ${\mathscr{P}}{\mathscr{T}}$ symmetry was also demonstrated in acoustics^42^ and electronic circuits^43^, and predicted in atomic Bose-Einstein condensates^44^, magnetism^45^, and chains of coupled pendula^46^.

The ${\mathscr{P}}{\mathscr{T}}$ symmetry, being a linear feature, is often combined with intrinsic nonlinearity of settings in which it is realized. Most typically, it is the Kerr nonlinearity of underlying optical media, which gives rise to nonlinear Schrödinger equations (NLSEs) with the cubic term and complex potentials, subject to the constraint given by Eq. (2). Such equations may generate ${\mathscr{P}}{\mathscr{T}}$-symmetric solitons, which were considered in many theoretical works^21,26–33^ (see also reviews^47,48^), and experimentally demonstrated too^38^. Although these works were chiefly dealing with one-dimensional (1D) models, stable ${\mathscr{P}}{\mathscr{T}}$-symmetric solitons were also predicted in some 2D settings^30,49–55^. A characteristic feature of ${\mathscr{P}}{\mathscr{T}}$-symmetric solitons is that, although existing in dissipative systems, they appear in continuous families, similar to their counterparts in conservative models^56^, while usual dissipative solitons exist as isolated solutions (*attractors*, if they are stable)^57,58^. The realization of the ${\mathscr{P}}{\mathscr{T}}$ symmetry in 2D geometry may provide essential extension of the above-mentioned applications, such as the unidirectional transmission, enhanced absorption, and lasing for broad optical beams.

Solitons are also vulnerable to destabilization via the ${\mathscr{P}}{\mathscr{T}}$-symmetry breaking at the critical value of the gain-loss coefficient^59^. Nevertheless, it was found that, in some settings, the solitons’ ${\mathscr{P}}{\mathscr{T}}$ symmetry can be made *unbreakable*, extending to arbitrarily large values of the strength of the model’s imaginary potential^60–62^, see also a brief review of the unbreakability concept in^63^. The particular property of these models is that self-trapping of solitons is provided not by the self-focusing sign of the nonlinearity, but by the defocusing sign, with the coefficient in front of the cubic term growing fast enough from the center to periphery. In the absence of gain and loss, this scheme of stable self-trapping was elaborated for 1D, 2D, and 3D bright solitons^64–69^. It is essential to stress that such models are *nonlinearizable*, which means that decaying tails of solitons are determined by the full nonlinear equation. In other words, the models have no linear spectrum, the spectrum of eigenstates being represented by nonlinear self-trapped modes (solitons). Accordingly, the models elaborated in refs ^60–62^ realize the ${\mathscr{P}}{\mathscr{T}}$ symmetry in a sense different from that defined in the usual systems—not in terms of the linear spectrum, which does not exist in this case, but in the form of stable families of complex-valued solitons with real propagation constants (eigenvalues), which exist in the presence of spatially odd imaginary potentials.

The present work introduces 2D models which maintain stable solitons, including (nearly) unbreakable ones, in the presence of the spatially growing self-defocusing nonlinearity and antisymmetric imaginary potentials, $iW(x,y)$ in Eq. (1). One model, with

3 $$W(x,y)={\gamma }_{0}x\,\exp \,(\,-\,\beta {r}^{2}),\,{r}^{2}={x}^{2}+{y}^{2},$$

where ${\gamma }_{0} > 0$ and $\beta \ge 0$ are constants, features the unbreakable or nearly unbreakable 2D ${\mathscr{P}}{\mathscr{T}}$ symmetry, represented by several species of families of stable solitons: single- and double-peak ones, as well as 2D solitons with embedded integer vorticity (topological charge), $m=1,2,3$. The second model, with

4 $$W(x,y)={\gamma }_{0}xy\,\exp \,(\,-\,\beta {r}^{2}),$$

is not, strictly speaking, a ${\mathscr{P}}{\mathscr{T}}$-symmetric one, but it is equally relevant for the realization in optics, and it shares basic manifestations of the ${\mathscr{P}}{\mathscr{T}}$ symmetry, maintaining families of single- and multi-peak solitons [featuring up to five peaks, in accordance with the structure of $W(x,y)$] and solitary vortices, also with $m=1,2,3$. The latter result is a contribution to the general topic of constructing models more general than the ${\mathscr{P}}{\mathscr{T}}$-symmetric ones with similar properties(including the case of the partial ${\mathscr{P}}{\mathscr{T}}$ symmetry^51^), which has been addressed in various settings^52–54,56,70–75^, see also review^47^.

In both models, universal analytical forms are obtained for tails of solitons, and full exact solutions are produced for particular species of single-peak solitons, with $\beta =0$ in Eqs (3) and (4). In the former case, the existence of the exact solitons at arbitrarily large values of *γ*_0_ in Eq. (3) explicitly demonstrates the unbreakability of the ${\mathscr{P}}{\mathscr{T}}$ symmetry. In the latter case two different families of exact solutions are found, which, however, exist only for ${\gamma }_{0}\le 2$ in Eq. (4) with $\beta =0$. In addition, an anisotropic version of the latter model gives rise to particular exact solutions for vortex solitons with topological charge $m=1$. Generic soliton families with $m=0,1,2,3$, which include the exact single-peak solutions as particular ones, are constructed in a numerical form in both models, and their stability is investigated numerically—both through computation of eigenvalues for small perturbations and by means of direct simulations.

## Results

### The models and analytical solutions for solitons

#### The underlying equations

The 1D NLSE for the amplitude of the electromagnetic field, *u*(*x*, *z*), with the local strength of the self-defocusing nonlinearity, ${\rm{\Sigma }}(x)$, growing from $x=0$ towards $x=\pm \,\infty$ faster than |*x*| (this condition is necessary for self-trapping imposed by the self-repulsion^64^), which is capable to maintain bright solitons with unbreakable ${\mathscr{P}}{\mathscr{T}}$ symmetry, is^60^

5 $$i\frac{\partial u}{\partial z}+\frac{1}{2}\frac{{\partial }^{2}u}{\partial {x}^{2}}-{\rm{\Sigma }}(x)|u{|}^{2}u=iW\,(x)u.$$

Here *z* and *x* are scaled propagation coordinate and transverse coordinate, in terms of the planar optical waveguide. In work^60^, the analysis was presented for a steep 1D modulation profile,

6 $${\rm{\Sigma }}(x)=(1+\sigma {x}^{2})\,\exp \,({x}^{2}),$$

with $\sigma \ge 0$, where coefficients equal to 1 may be fixed to these values by means of rescaling. The choice of this profile allows one to obtain a particular exact solution for solitons^64^. Of course, in a real physical medium the local strength of the nonlinearity, defined as per Eq. (6), cannot grow to infinitely large values at $|x|\to \infty$. However, in reality it is sufficient that it grows according to Eq. (6) to finite values, that correspond to |*x*| which is essentially larger than the width of the soliton created by this profile. The growth of ${\rm{\Sigma }}(x)$ may be safely aborted at still larger |*x*|^64^.

Further, the spatially-odd imaginary potential, which accounts for the ${\mathscr{P}}{\mathscr{T}}$-symmetric gain-loss profile (cf. Eq. (1)), was introduced in ref. ^60^ as

7 $$W(x)={\gamma }_{0}x\,\exp \,(\,-\,\beta {x}^{2}),$$

with ${\gamma }_{0} > 0$ and $\beta \ge 0$. In the case of the spatially uniform self-focusing cubic nonlinearity, the 1D imaginary potential in the form given by Eq. (7) was introduced in ref. ^76^.

Here, we aim to  define a 2D extension of the model, as the NLSE for the propagation of the electromagnetic field with amplitude *u*(*x*, *y*, *z*) in the bulk waveguide with transverse coordinates (*x*, *y*):

8 $$i\frac{\partial u}{\partial z}+\frac{1}{2}(\frac{{\partial }^{2}u}{\partial {x}^{2}}+\frac{{\partial }^{2}u}{\partial {y}^{2}})-{\rm{\Sigma }}(r)|u{|}^{2}u=iW\,(x,y)\,u,$$

where $r\equiv \sqrt{{x}^{2}+{y}^{2}}$ is the radial coordinate, and the nonlinearity-modulation profile is chosen similar to its 1D counterpart (6):

9 $${\rm{\Sigma }}(r)=(1+\sigma {r}^{2})\,\exp \,({r}^{2})$$

with $\sigma \ge 0$. Further, we consider two different versions of the 2D imaginary potential. First, it is a ${\mathscr{P}}{\mathscr{T}}$-symmetric one given by Eq. (3). The other imaginary potential, defined as per Eq. (4), is not ${\mathscr{P}}{\mathscr{T}}$-symmetric, because the ${\mathscr{P}}$ transformation, $(x,y)\to (\,-\,x,-\,y)$, does not reverse the sign of *W*(*x*, *y*), in this case. However, in terms of the implementation in optics the gain-loss distribution corresponding to Eq. (4) is as relevant as that defined by Eq. (7), and, as mentioned above, properties of solitons in models which are akin to ${\mathscr{P}}{\mathscr{T}}$-symmetric ones is a subject of considerable interest.

Stationary states with a real propagation constant, *k*, are looked for as solutions to Eq. (8) in the form of

10 $$u\,(x,y)=\exp \,(ikz)\,U\,(x,y),$$

with complex function *U*(*x*, *y*) satisfying the following equation:

11 $$kU=\frac{1}{2}\,(\frac{{\partial }^{2}U}{\partial {x}^{2}}+\frac{{\partial }^{2}U}{\partial {y}^{2}})-{\rm{\Sigma }}(r)|U{|}^{2}U-iW\,(x,y)\,U.$$

#### Asymptotic solutions

As mentioned above, Eqs (8) and (11) are *nonlinearizable*, i.e., they cannot be characterized by a linear spectrum. Indeed, straightforward analysis of Eq. (11) demonstrates that it may produce localized solutions (solitons), with tails decaying at $r\to \infty$ according to an asymptotic expression which is determined by the full nonlinear equation, rather than by its linearization. For the ${\mathscr{P}}{\mathscr{T}}$-symmetric imaginary potential (3) with $\beta =0$, it is

12 $${U}_{{\rm{asympt}}}\,(x,y)=\frac{1}{\sqrt{2\sigma }}\,\exp \,(-\frac{1}{2}{r}^{2}-i{\gamma }_{0}x),$$

provided that $\sigma \ne 0$. In the case case of $\sigma =0$, this asymptotic solution is replaced by

13 $${U}_{{\rm{asympt}}}\,(x,y)=\frac{r}{\sqrt{2}}\,\exp \,(-\frac{1}{2}{r}^{2}-i{\gamma }_{0}x).$$

Note that asymptotic solutions given by Eqs (12) and (13) exist at *arbitrarily large γ*_0_, suggesting the *unbreakability* of the ${\mathscr{P}}{\mathscr{T}}$ symmetry in this case, as corroborated by exact solution (19) produced below.

The imaginary potential defined by Eq. (4) with $\beta =0$ produces the following result:

14 $${U}_{{\rm{asympt}}}\,(x,y)=\sqrt{\frac{1-{({\gamma }_{0}/2)}^{2}}{2\sigma }}\,\exp \,(-\frac{1}{2}{r}^{2}-\frac{1}{2}i{\gamma }_{0}xy),$$

for $\sigma \ne 0$, and if $\sigma =0$, the result is

15 $${U}_{{\rm{asympt}}}\,(x,y)=\sqrt{\frac{1-{({\gamma }_{0}/2)}^{2}}{2}}r\,\exp \,(-\frac{1}{2}{r}^{2}-\frac{1}{2}i{\gamma }_{0}xy).$$

On the contrary to the the above asymptotic solutions, given by Eqs (12) and (13), which are available for arbitrarily large *γ*_0_, their counterparts produced by Eqs (14) and (15) exist only at ${\gamma }_{0} < 2$, i.e., if the gain-loss coefficient is not too large.

It is relevant to stress the *universal character* of all asymptotic approximations given by Eqs (12–15): they depend solely on coefficients *σ* and *γ*_0_ of the underlying model, and, unlike the commonly known asymptotic forms of solitons in usual systems, do not depend on the propagation constant, *k*. The single exception is presented by exact solution Eq. (18) given below, whose asymptotic form (actually coinciding with the exact soliton solution, in that case) explicitly depends on *k*, but this happens solely for specially chosen parameters given by Eq. (17). In the generic case, a dependence on *k* appears in the next-order correction to the shape of the asymptotic tail. In particular, the correction to the tails given by Eqs (12) and (13) are

16 $$\delta {U}_{{\rm{asympt}}}\,(x,y)=-\,(k/{r}^{2})\,{U}_{{\rm{asympt}}}\,(x,y).$$

Furthermore, for more complex solutions, such as multi-peak solitons and solitary vortices, as well as for higher-order radial states of the single-peak solitons, which are produced below in the numerical form, the asymptotic expression at large *r* is exactly the same as given by Eqs (12–15).

#### Exact solutions for single-peak solitons

Precisely at the above-mentioned critical value ${\gamma }_{0}=2$, the asymptotic solutions (14) and (15) vanish. However, in the special case,

17 $$\sigma =0,\,{\gamma }_{0}=2,\,\beta =0,$$

the vanishing asymptotic solution given by Eq. (15) is replaced by a different one, which, as can be easily checked, is an *exact solution* to Eq. (11) (not just an asymptotic approximation valid at large *r*),

18 $${({U}_{{\rm{exact}}}^{(xy)})}_{{\gamma }_{0}=2}=\sqrt{-(1+k)}\,\exp \,(-\frac{1}{2}{r}^{2}-\frac{1}{2}i{\gamma }_{0}xy).$$

It exists, as the continuous family, at all values of $k < -\,1$.

**Figure 1:**
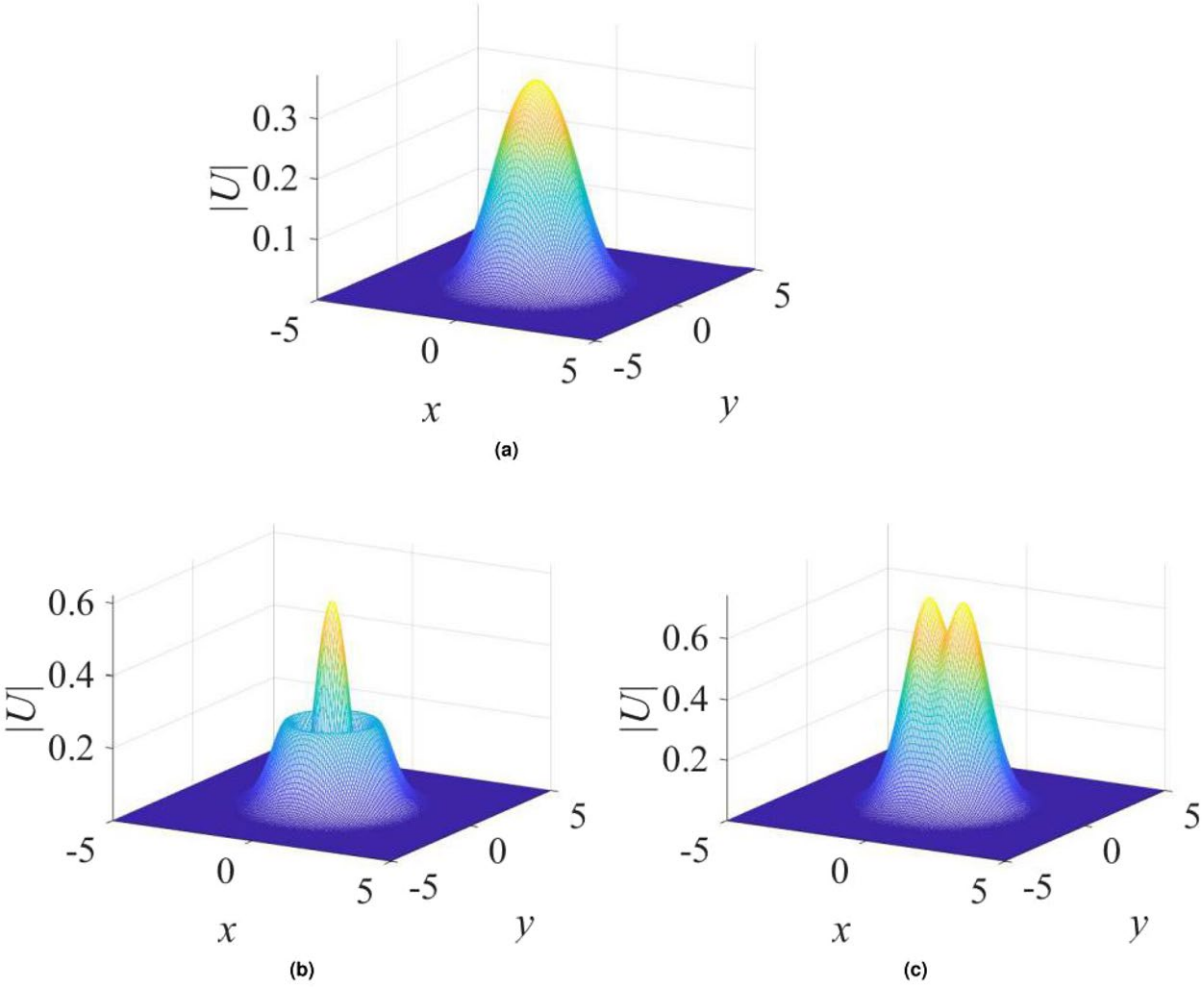
Typical examples of stable solitons produced by the model with the ${\mathscr{P}}{\mathscr{T}}$-symmetric imaginary potential defined by Eq. (3). (**a**) A fundamental single-peak soliton for ${\gamma }_{0}=1.2$ in Eq. (3) and propagation constant $k=-\,3.2$ in Eq. (10). (**b**) A higher-order radial state of the single-peak soliton for ${\gamma }_{0}=0.2$ and $k=-\,4$. (**c**) A double-peak soliton for ${\gamma }_{0}=1.4$ and $k=-\,4$. In all the cases, $\sigma =1$ and $\beta =0$ are fixed in Eqs (3) and (9).

**Figure 2:**
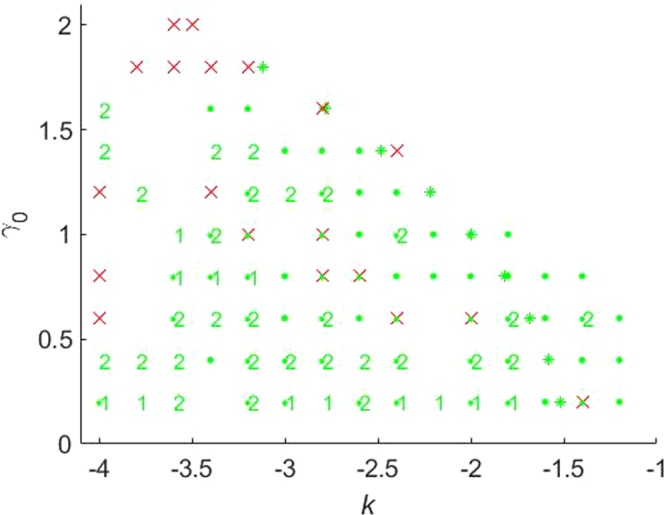
The stability map for the ${\mathscr{P}}{\mathscr{T}}$-symmetric solitons maintained by imaginary potential (3), in the case of $\sigma =1$ and $\beta =0$ in Eqs (3) and (9). Stable fundamental single-peak solitons are marked by green dots. All unstable solitons are marked by red crosses, irrespective of their structure. Exact soliton solutions, given by Eqs (19) and (20), are indicated by green stars (except for one at ${\gamma }_{0}=2$, which is designated by the red cross, as the exact solutions are unstable at ${\gamma }_{0}\ge 2$). Green numbers ≥2 in this figure and below denote stable solitons with the same number of peaks. Further, green numbers 1 label stable single-peak solitons with the higher-order radial structure, as in Fig. 1(b). Green numbers 1 or 2, placed close to green dots, imply bistability, i.e., coexistence of stable fundamental single-peak solitons and stable higher-order or double-peak ones. Red crosses placed on top of green dots imply coexistence of fundamental single-peak solitons with some unstable mode. Soliton solutions were not found in white areas.

**Figure 3:**
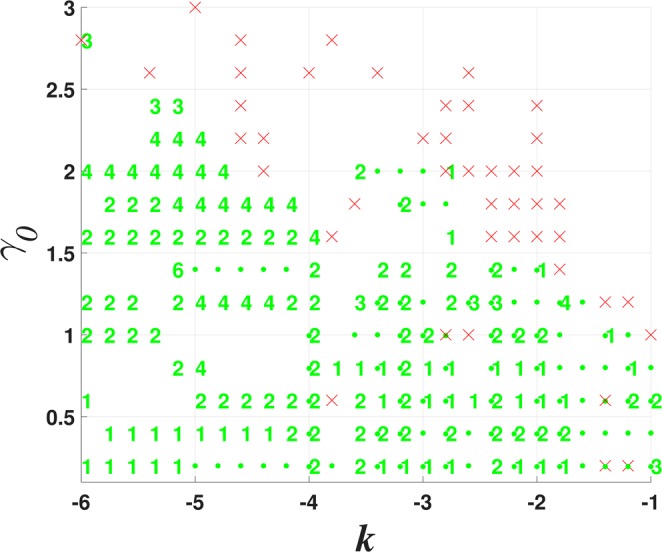
The same as in Fig. 2, but for $\beta =0.2$ in Eq. (3), i.e., with the gain-loss term subject to weak spatial confinement. In this case, there are no exact solitons solutions, while the asymptotic solution for the tails is given by Eq. (12) with ${\gamma }_{0}=0$ (the confinement eliminates *γ*_0_ from the asymptotic solution).

Further, Eq. (11) which includes the ${\mathscr{P}}{\mathscr{T}}$-symmetric imaginary potential Eq. (3), with $\beta =0$, gives rise to an exact solution at a special value ${k}_{0}^{(x)}$ of the propagation constant:

19 $${U}_{{\rm{exact}}}^{(x)}=\frac{1}{\sqrt{2\sigma }}\,\exp \,(-\frac{1}{2}{r}^{2}-i{\gamma }_{0}x),$$

20 $${k}_{0}^{(x)}=-\,(1+\frac{{\gamma }_{0}^{2}}{2}+\frac{1}{2\sigma }),$$

which exists at all values of coefficients *γ*_0_ and *σ*, except for $\sigma =0$. In other words, at $k={k}_{0}^{(x)}$ the asymptotic approximation Eq. (12) is tantamount to the exact solution. This solution features the *unbreakable* ${\mathscr{P}}{\mathscr{T}}$ symmetry, as it persists at arbitrarily large values of the gain-loss coefficient, *γ*_0_. Moreover, although Eq. (19) yields the exact solution at the single value of the propagation constant, given by Eq. (20), which is embedded in a generic family of numerically found fundamental solitons, as demonstrated below in Figs 1, 2 and 3, the entire family asymptotically shrinks to the exact solution in the limit of large *γ*_0_. Indeed, it is easy to find that, for ${\gamma }_{0}^{2}\gg 1$ and a relatively small deviation of the propagation constant from the special value (20), $|\delta k|\equiv |k-{k}_{0}^{(x)}|\ll {\gamma }_{0}^{2}$, the fundamental soliton is

21 $${U}_{{\rm{approx}}}^{(x)}\approx \frac{1}{\sqrt{2\sigma }}\,\exp \,[-\frac{1}{2}({r}^{2}+\frac{\delta k}{{\gamma }_{0}^{2}}{x}^{2})-i({\gamma }_{0}-\frac{\delta k}{{\gamma }_{0}})\,x],$$

featuring weak anisotropy of the shape, $|{U}_{{\rm{approx}}}^{(x)}(x,y)|$.

Next, Eq. (11) with the imaginary potential taken as per Eq. (4) with $\beta =0$, and with $\sigma \ne 0$ in the nonlinearity-modulation profile (9), gives rise to the following exact solution, at the respective single value of *k*:

22 $${({U}_{{\rm{exact}}}^{(xy)})}_{{\gamma }_{0} < 2}=\sqrt{\frac{1-{({\gamma }_{0}/2)}^{2}}{2\sigma }}\,\exp \,(-\frac{1}{2}{r}^{2}-\frac{1}{2}i{\gamma }_{0}xy),$$

23 $${k}_{0}^{(xy)}=-\,[1+\frac{1}{2\sigma }(1-{(\frac{{\gamma }_{0}}{2})}^{2})].$$

In this case too, the asymptotic approximation Eq. (14) becomes identical to the exact solution at $k={k}_{0}^{(xy)}$, both existing at ${\gamma }_{0} < 2$, on the contrary to exact solution (19), which exists at all values of *γ*_0_.

Thus, the models considered here do not have the linear spectrum. Instead of it, they are characterized by spectra (families) of self-trapped nonlinear solutions (solitons). The radical change of the concept of the system’s spectrum implies a respective change in the concept of the ${\mathscr{P}}{\mathscr{T}}$ symmetry, which now applies not to the set of eigenvalues of the linearized system, but directly to families of nonlinear states. Lastly, it is worthy to note that all the asymptotic and exact solutions produced above, including the first correction (16) to the asymptotic tails, feature isotropic shapes of |*U*(*x*, *y*)|, although the imaginary potentials Eqs (3) and (4) are obviously anisotropic.

#### Exact solutions for elliptic vortices in an anisotropic model

In addition to 2D fundamental solitons, similar to the exact ones presented here, we also address below, by means of numerical methods, solitons with embedded vorticities, $m=1,2,3\ldots$. A challenging issue is to seek for exact solutions for vortex solitons. Such solutions can be found in the case of imaginary potential Eq. (4) with $\beta =0$, in a more general *anisotropic* version of the nonlinearity-modulation profile in Eq. (8) with $\sigma =0$, namely,

24 $${\rm{\Sigma }}\,(x,y)=\exp \,({x}^{2}+g{y}^{2}),$$

where positive $g\ne 1$ accounts for the ellipticity of the modulation profile. Then, an exact solution for elliptically deformed vortex solitons with $m=1$ is given by the following ansatz [cf. Eq. (22)]:

25 $$U(x,y)={U}_{0}\,(x+iby)\,\exp \,(-\frac{1}{2}\,({x}^{2}+g{y}^{2})-iaxy),$$

where real $b\ne 1$ accounts for the ellipticity of the soliton’s phase field, and *a* is another real constant. The substitution of this ansatz and expressions Eqs (4) and (24) (with $\beta =0$) in the accordingly modified Eq. (11) leads to the following relations between parameters of the ansatz:

26 $$\begin{array}{rcl}(1+g)a & = & -\,{\gamma }_{0},\\ (g-1)b-(1+{b}^{2})a & = & 0,\\ {b}^{2}(1-{a}^{2})+{a}^{2} & = & {g}^{2},\end{array}$$

supplemented by expressions for the propagation constant and soliton’s amplitude:

27 $$k=-\,(3/2+g/2+ab),\,{U}_{0}^{2}=(1-{a}^{2})/2.$$

The system of three equations (26) for two free parameters *a* and *b* demonstrates that the exact vortex solution is a nongeneric one, as it may exist only if an additional constraint, which can be derived by eliminating *a* and *b* in Eq. (27), is imposed on parameters *g* and *γ*_0_:

$${({g}^{2}-1)}^{2}\,[{g}^{2}\,{(g+1)}^{2}-{\gamma }_{0}^{2}]\,[{(g+1)}^{2}-{\gamma }_{0}^{2}]={\gamma }_{0}^{2}\,{[({g}^{2}+1){(g+1)}^{2}-2{\gamma }_{0}^{2}]}^{2}.$$

In the isotropic model, with $g=1$, Eq. (26) has no nontrivial solutions. However, they can be found for $g\ne 1$. A particular example is

$$\begin{array}{rcl}b & = & 1/\sqrt{2}\approx 0.707\,1,\,a=-\,(3-\sqrt{5})/(4\sqrt{2})\approx -\,0.1351,\\ {U}_{0} & = & \sqrt{3(3+\sqrt{5})}/(4\sqrt{2})\approx 0.7006,\end{array}$$

which is a valid solution at $g=(3\sqrt{5}-1)/8\approx 0.7135$ and ${\gamma }_{0}=(3+\sqrt{5})/(16\sqrt{2})\approx 0.2314$. This value of *g* corresponds to eccentricity $e\equiv \sqrt{1-g}=\sqrt{(9-3\sqrt{5})/8}\approx 0.5352$ of the elliptic profile in Eq. (24).

Numerical results are reported below for the isotropic model, while the anisotropic one should be a subject for separate consideration.

### Numerical results for zero-vorticity solitons

#### The ${\mathscr{P}}{\mathscr{T}}$-symmetric imaginary potential (3): single- and double-peak solitons

The isolated exact solution of the model with the ${\mathscr{P}}{\mathscr{T}}$-symmetric gain-loss distribution, given by Eqs (19) and (20), can be embedded in a continuous family of solitons, produced by a numerical solution of Eq. (11), with ${\rm{\Sigma }}(r)$ and *γ*(*x*) taken as per Eqs (3) and (9). The appropriate numerical algorithm is the Newton conjugate gradient method ^77^, which is briefly outlined in section Method below. The stability of the stationary states was identified by numerical computation of eigenvalues of small perturbations, using linearized Eq. (32) for perturbations around the stationary solitons. Finally, the stability predictions, produced by the eigenvalues, were verified by simulations of the perturbed evolution of the solitons (some technical details are reported elsewhere^63^).

It is relevant to stress that the convergence of the algorithm which produces stationary states depends on appropriate choice of the initial guess. While stationary modes were not found in “ holes” appearing in stability charts which are displayed below in Figs 2, 3, 7, 10, 11, 12, 16 and 17, it is plausible that stationary solutions exist in the holes too, being, however, especially sensitive to the choice of the input. On the other hand, the intricate alternation of stability and instability spots, which is also observed in the charts, is a true peculiarity of the present model. Moreover, genuine structure of the stability charts may be fractal, but analysis of this possibility is beyond the scope of the present work.

Generic examples of numerically found *stable* solitons with single- and double-peak shapes are displayed in Fig. 1. Note that the double-peak modes have their two maxima separated in the direction of *x*, in accordance with the anisotropic shape of the imaginary potential in Eq. (3). As concerns single-peak modes, two different varieties of stable ones were found: fundamental solitons, with the shape similar to that of the exact solution given by Eqs (19) and (20) [see Fig. 1(a)], and higher-order states with a radial ring surrounding the central peak, see Fig. 1(b). It is worthy to note that, unlike many other models, where higher-order radial states are unstable^78–83^, they are stable in the present case. Note also that shapes of both species of the single-peak solitons, fundamental and higher-order ones, seem isotropic in terms of |*U*(*x*, *y*)|, similar to exact solution (19). The isotropy is obviously broken by double-peak modes, see Fig. 1(c).

Results of the stability analysis, based on the computation of perturbation eigenvalues, are summarized in the stability map in the plane of (*k*, *γ*_0_) [the soliton’s propagation constant and strength of the gain-loss term in Eq. (3)], for $\beta =0$ and $\beta =0.2$ in Figs 2 and 3, respectively. Several noteworthy features are revealed by these plots. First, it is worthy to note significant stability areas for both the double-peak and higher-order single-peak ${\mathscr{P}}{\mathscr{T}}$-symmetric solitons in Figs 2 and 3. Further, bistability is observed at many points, in the form of coexisting stable fundamental and double-peak solitons, or fundamental and higher-order radial states of single-peak ones. As concerns the possibility of maintaining the unbreakable ${\mathscr{P}}{\mathscr{T}}$ symmetry, Fig. 2 demonstrates shrinkage of the existence and stability regions of the modes with the increase of *γ*_0_ at $\beta =0$ to the exact soliton solution given by Eqs (19) and (20), in agreement with the trend represented by approximate solution (21). Eventually, the exact solution loses its stability at ${\gamma }_{0}\ge 2$. On the other hand, the introduction of a relatively weak confinement of the gain-loss term, with $\beta =0.2$ in Eq. (3), demonstrates that the ${\mathscr{P}}{\mathscr{T}}$ symmetry remains unbreakable in Fig. 3, where both the existence and stability regions extend in the direction of large values of −*k* and *γ*_0_, without featuring any boundary.

As concerns unstable solitons, they typically blow up in the course of the evolution, see an example below in Fig. 18. Although it shows the blowup of a vortex soliton, the instability development of zero-vorticity ones is quite similar.

The stability charts, drawn in Figs 2 and 3 for $\sigma =1$ in Eq. (9), are similar to their counterparts produced at other values of *σ*, including $\sigma =0$, when the exact solution given by Eqs (19) and (20) does not exist, while the asymptotic form of the solitons’ tails is given by Eq. (13).

#### The imaginary potential (4): single- and multi-peak solitons

A drastic difference revealed by the stability analysis of the model based on Eqs (4), (8) and (9) is that the respective exact solutions, given by Eq. (18) for the special case (17), and by Eqs (22) and (23) for $\sigma  > 0$, $\beta =0$ and arbitrary *γ*_0_, are completely unstable, on the contrary to the stability of the exact solutions in the case of the ${\mathscr{P}}{\mathscr{T}}$-symmetric imaginary potential Eq. (3) (at ${\gamma }_{0} < 2$). Furthermore, all numerical solutions found in the full 2D model with $\beta =0$ in Eq. (4) are unstable too. The stabilization in this model is provided by $\beta  > 0$, i.e., by imposing the spatial confinement on the gain-loss term in Eq. (4). For fixed *σ*, there is a minimum value *β*_min_ of *β* which secures the stabilization. For instance, we have concluded that the solitons may be stable in the model with $\sigma =1$ in Eq. (9) at $\beta \ge {\beta }_{{\rm{\min }}}\approx 0.2$ in Eq. (4), still being completely unstable, e.g., at $\beta =0.1$.

**Figure 4:**
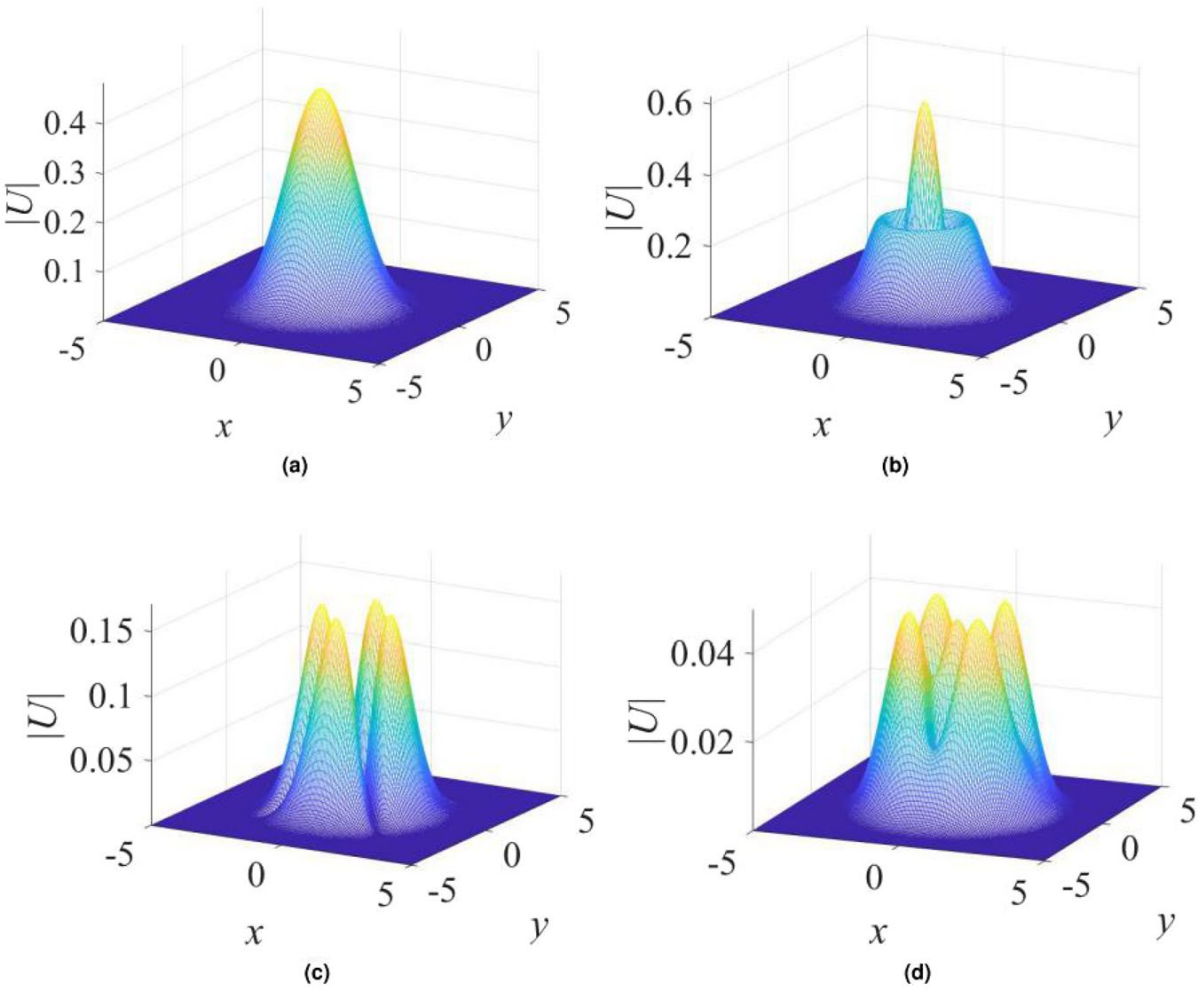
Examples of stable single- and multi-peak ${\mathscr{P}}{\mathscr{T}}$-symmetric solitons, found in the model based on Eqs (4) and (9), with $\sigma =1$ and (**a**) $\beta =0.5$, ${\gamma }_{0}\mathrm{=1}$, $k=-\,1$; (**b**) $\beta =0.5$, ${\gamma }_{0}=0.2$, $k=-\,4$; (**c**) $\beta =0.2$, ${\gamma }_{0}=1.4$, $k=-\,2.8$; (**d**) $\beta =0.5$, ${\gamma }_{0}=0.4$, $k=-\,1.8$.

As mentioned above, the steep growth of ${\rm{\Sigma }}(r)$ in Eq. (9) cannot extend to infinity, it being sufficient to maintain the adopted profile of ${\rm{\Sigma }}(r)$ on a scale which is essentially larger than a characteristic size of solitons supported by this profile. The same pertains to the linear growth of the imaginary potential at large |*x*| in Eq. (3): in reality, it should not continue at distances much larger than the size of the stable solitons considered in the previous section. However, the presence of *β*_min_ implies that the corresponding “tacit” confinement of *γ*(*x*, *y*) in Eq. (4) is not sufficient to produce stable 2D solitons. At $\beta  > {\beta }_{{\rm{\min }}}$, the numerical solution generates stable fundamental single-peak solitons and their higher-order radial counterparts with isotropic shapes of |*U*(*x*, *y*)|, as shown in Fig. 4(a,b). Further, stable multi-peak solitons are found too. Due to the 2D structure of the imaginary potential (4), they feature a four- or five-peak structure, built along both the *x* and *y* axes, as shown in Fig. 4(c,d), instead of the uniaxial double-peak modes supported by the quasi-1D imaginary potential (3), cf. Fig. 1(c).

**Figure 5:**
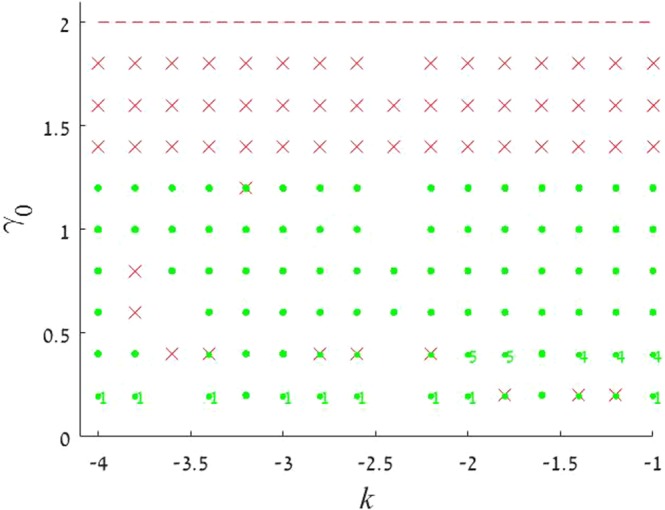
The stability chart, defined as in Figs 2 and 3, but for the model including imaginary potential (4), with $\sigma =1$ and $\beta =0.5$ in Eqs (4) and (9). As indicated by the upper dashed red curve, no solitons were found at ${\gamma }_{0}\ge 2$, where the exact solution given by Eq. (22) does not exist either.

A typical stability chart for the 2D solitons generated by the model with $\beta  > {\beta }_{{\rm{\min }}}$ is displayed in Fig. 5. It features bistability between the fundamental single-peak solitons and the higher-order ones, or four- and five-peak complexes, in a relatively small region of the (*k*, *γ*_0_) plane, at sufficiently small values of *γ*_0_. Figure 5 clearly shows that no solitons were found at ${\gamma }_{0}\ge 2$, this restriction coinciding with that for the exact solution given by Eqs (22) and (23). Thus, unlike the ${\mathscr{P}}{\mathscr{T}}$-symmetric imaginary potential (3), the model based on potential (4) does not produce unbreakable soliton families.

### Vortex solitons

Soliton solutions of Eq. (11) with embedded vorticity were found numerically by means of the above-mentioned Newton conjugate gradient method, initialized by the ansatz with integer vorticity $m\ge 1$ added to the previously found 2D stationary solutions of Eq. (11):

28 $$U\,(x,y)\to U\,(x,y)\,{r}^{m}\,\exp (im\theta )\equiv U\,(x,y)\,{(x+iy)}^{m},$$

where (*r*, *θ*) are the polar coordinates. The stability of resulting vortex solitons was again analyzed through the computation of eigenvalues for modes of small perturbations around the vortex states, see Eq. (32), and then verified by direct simulations.

#### Vortex solitons in the case of the ${\mathscr{P}}{\mathscr{T}}$-symmetric imaginary potential

**Figure 6:**
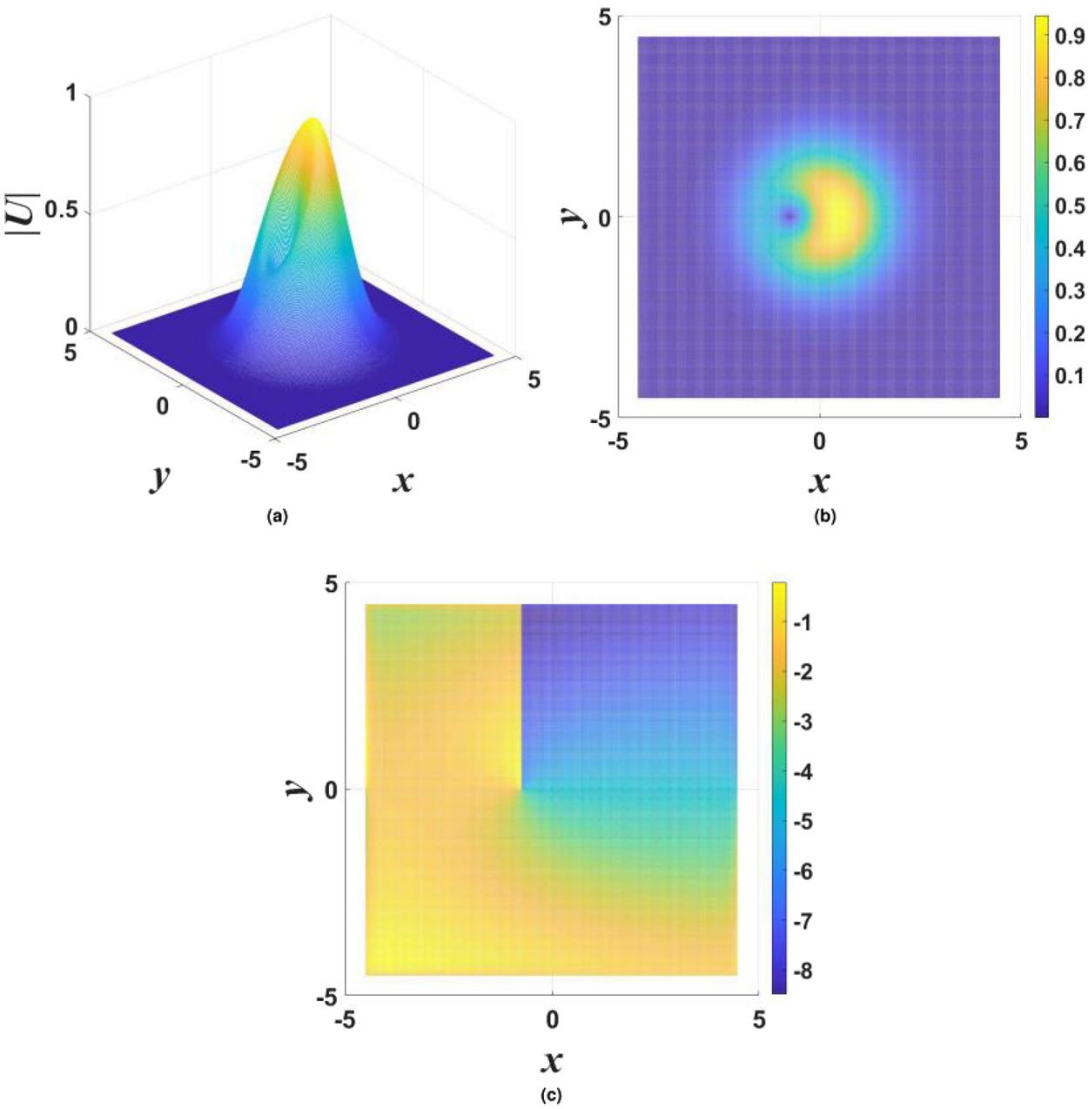
Three-dimensional (**a**) and top-view (**b**) shapes of |*U*(*x*, *y*)| for a typical stable vortex soliton with $m=1$, supported by the ${\mathscr{P}}{\mathscr{T}}$-symmetric imaginary potential (3) with ${\gamma }_{0}=0.6$, $\beta =0$, and $\sigma =0$ in Eq. (9), the propagation constant being $k=-\,3$. Panel (c) displays the phase structure of the vortex.

**Figure 7:**
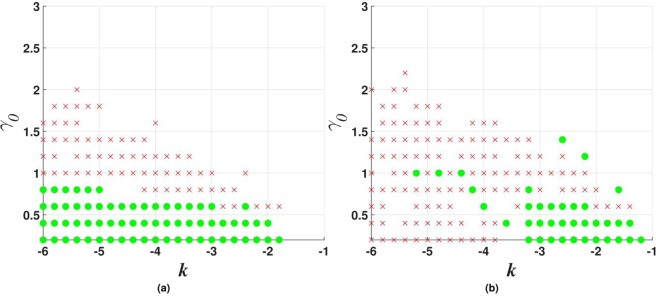
Stability charts for vortex solitons with topological charge $m=1$ in the model including the ${\mathscr{P}}{\mathscr{T}}$-symmetric imaginary potential (3) with $\beta =0$, and $\sigma =0$ or 1 in Eq. (9), in panels (a and b) panels, respectively. Green circles and red crosses denote stable and unstable vortex solitons, respectively. The same notation is used below in other stability charts for vortex solitons.

In the framework of the model with imaginary potential (3), stable vortex solitons were found in the case of $\beta =0$ (no gain-loss confinement) with $m=1$, while vortices with $m\ge 2$ do not exist or are unstable. An example of stable vortices is shown in Fig. 6, and the respective stability charts for different values of *σ* in Eq. (9) are presented in Fig. 7. The strongly anisotropic shape of the vortex is a consequence of the anisotropy of the underlying imaginary potential (3).

**Figure 8:**
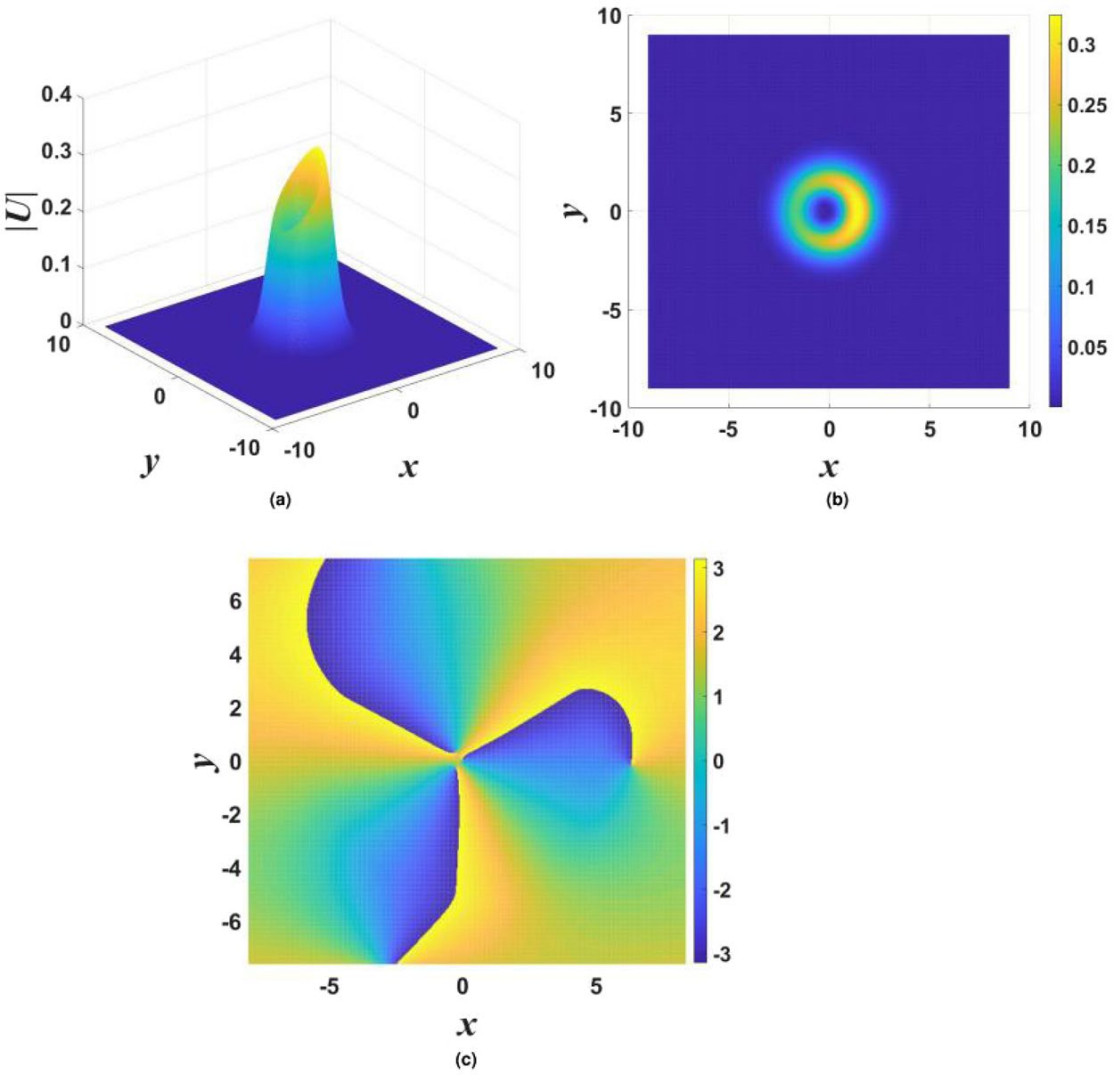
The same as in Fig. 6, but for stable vortex soliton with $m=3$ and parameters ${\gamma }_{0}=0.8$, $\beta =0.5$, $\sigma =0$, $k=-\,4$.

The introduction of the confinement of the gain and loss in Eq. (3) (in particular, setting $\beta =0.5$) makes it possible to construct stable vortex solitons with higher vorticities, corresponding to $m > 1$ in Eq. (28). An example of a stable vortex with $m=3$ is shown in Fig. 8.

**Figure 9:**
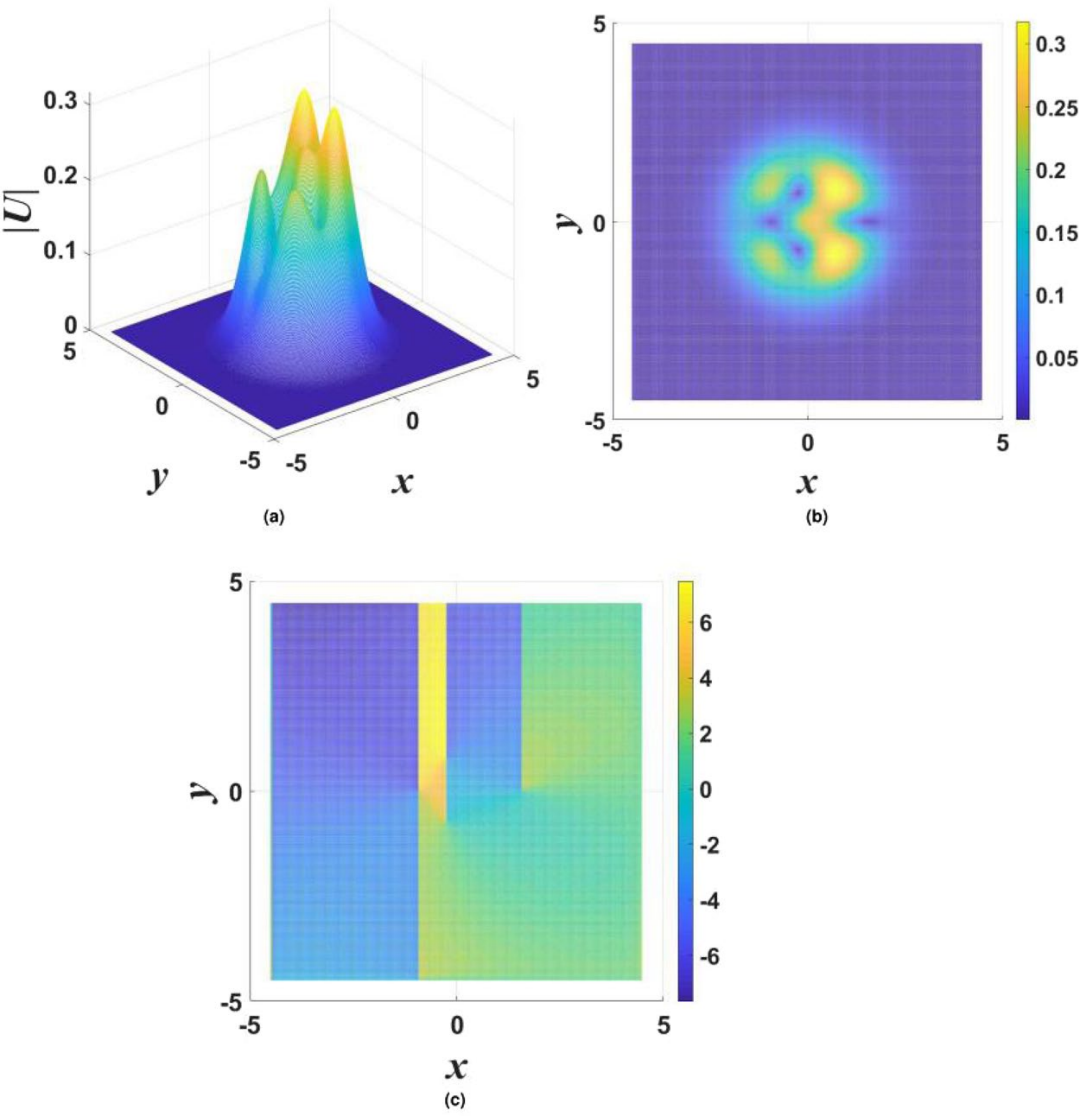
The same as in Fig. 6, but for a case when the stable vortex soliton with $m=1$, featuring a complex shape, is created, the parameters in Eqs (3) and (9) being ${\gamma }_{0}=0.4$, $\beta =0$, and $\sigma =1$. The propagation constant is $k=-\,3.6$.

In most cases, stable vortices generated by input (28) from double-peak stationary solutions have the same shape as those originating from their single-peak counterparts. However, in few cases the application of the lowest vorticity, with $m=1$ in Eq. (28), to the double-peak input leads to the creation of stable vortex solitons with a complex shape, see an example in Fig. 9.

**Figure 10:**
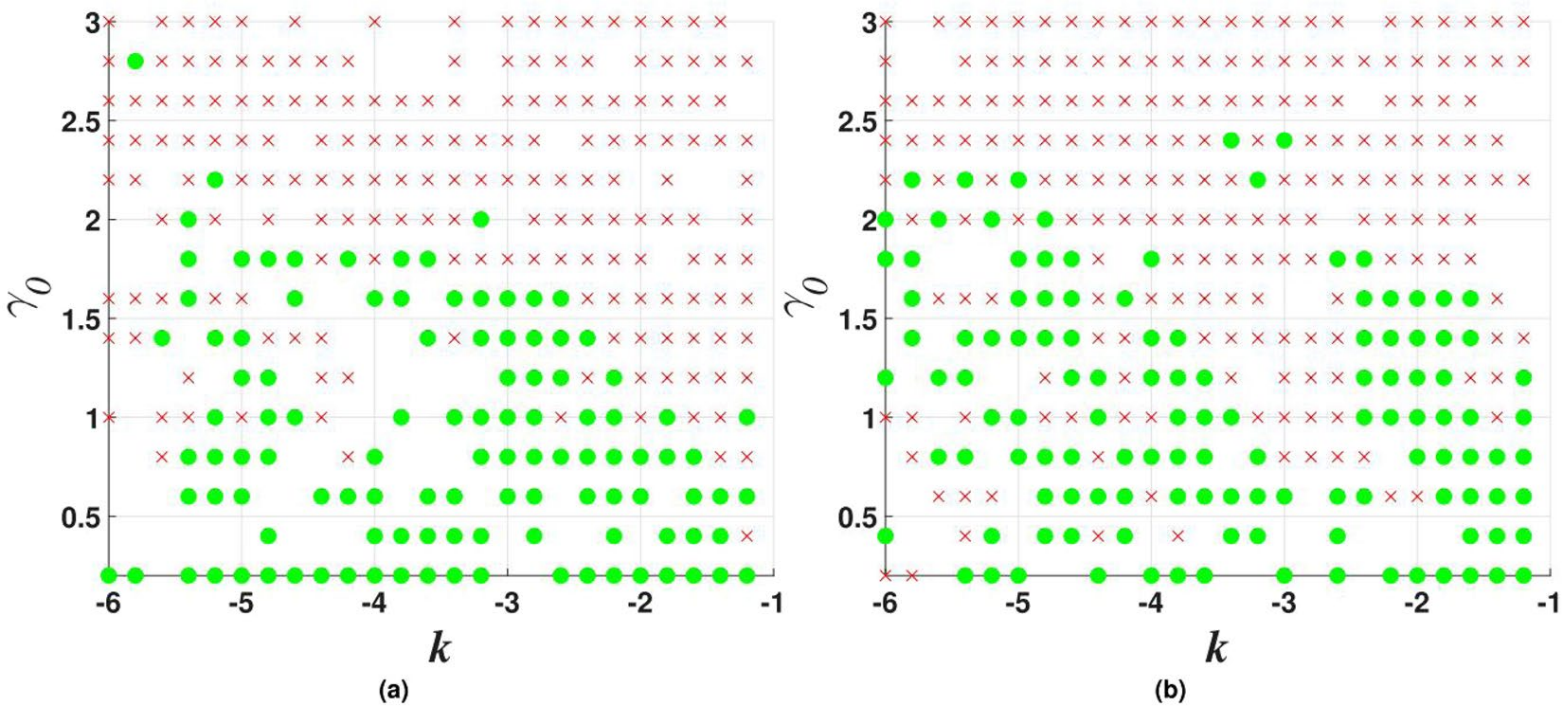
Stability charts for solitons with vorticity $m=1$ in the case of the ${\mathscr{P}}{\mathscr{T}}$-symmetric imaginary potential (3) with $\beta =0.5$, and $\sigma =0$ or 1 in Eq. (9), in panels (a and b), respectively.

**Figure 11:**
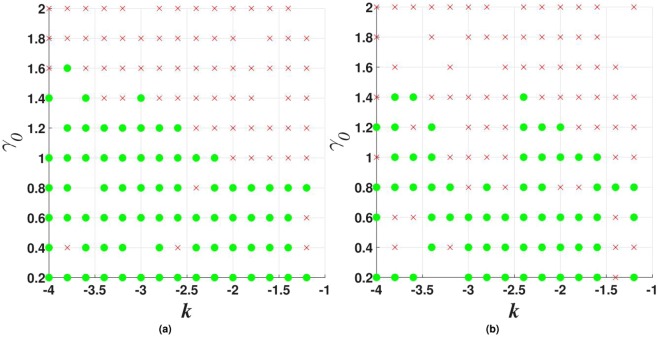
The same as in Fig. 10 (stability charts) but for vortex solitons with $m=2$.

**Figure 12:**
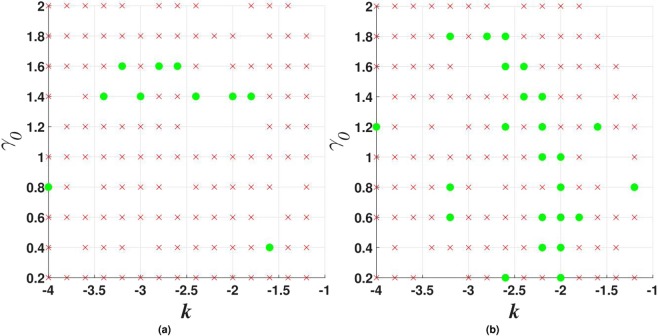
The same as in Fig. 10, but for vorticity $m=3$.

Stability charts for the vortex solitons with *m* = 1, 2, and 3, supported by the ${\mathscr{P}}{\mathscr{T}}$-symmetric imaginary potential which is subject to the spatial confinement, with $\beta =0.5$ in Eq. (3), are shown in Figs 10, 11 and 12. While the stability area shrinks with the increase of *m*, a few stable isolated modes were found even for $m=4$ (not shown here). The comparison of Figs 7 and 10 shows that the introduction of the spatial confinement of the gain-loss profile helps to expand the stability area for $m=1$ towards larger values of *γ*_0_, thus upholding the trend to observe the unbreakable ${\mathscr{P}}{\mathscr{T}}$ symmetry in this 2D model. In direct simulations, the evolution of unstable vortex modes leads towards the blowup, via their fusion into a single peak, similar to what is displayed below in Fig. 18.

#### Vortex solitons in the model with imaginary potential (4)

**Figure 13:**
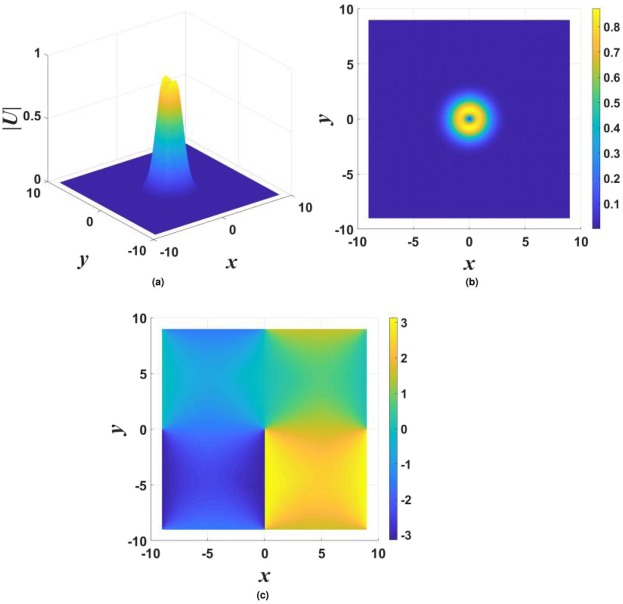
The same as in Fig. 6, but for the stable vortex soliton with $m=1$ in the case of imaginary potential given by Eq. (4), with ${\gamma }_{0}=0.4$, $\beta =0.5$, $\sigma =0$, and propagation constant $k=-\,3.4$.

**Figure 14:**
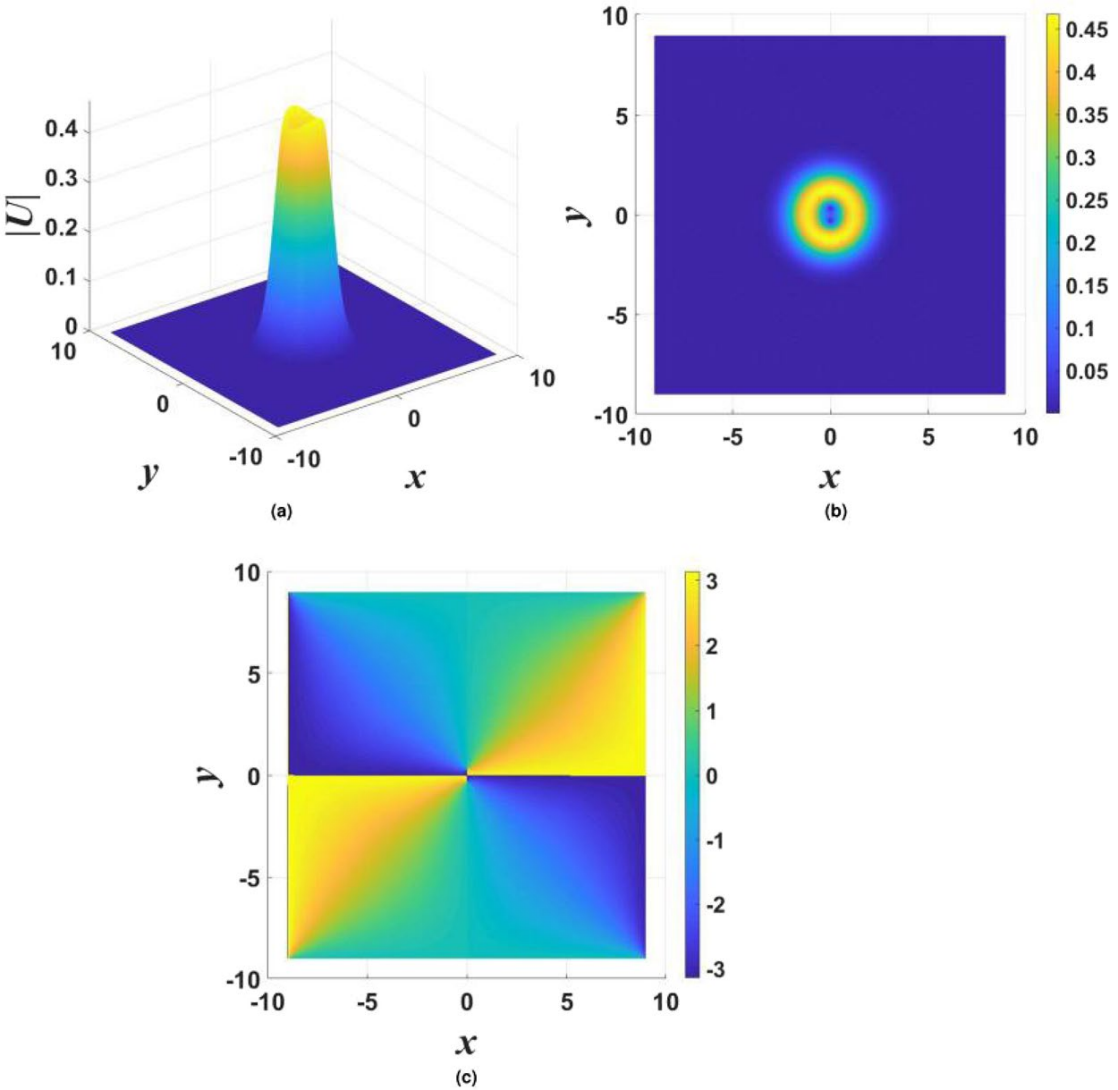
The same as in Fig. 13, but for stable vortex solitons with $m=2$ and $k=-\,3.6$.

**Figure 15:**
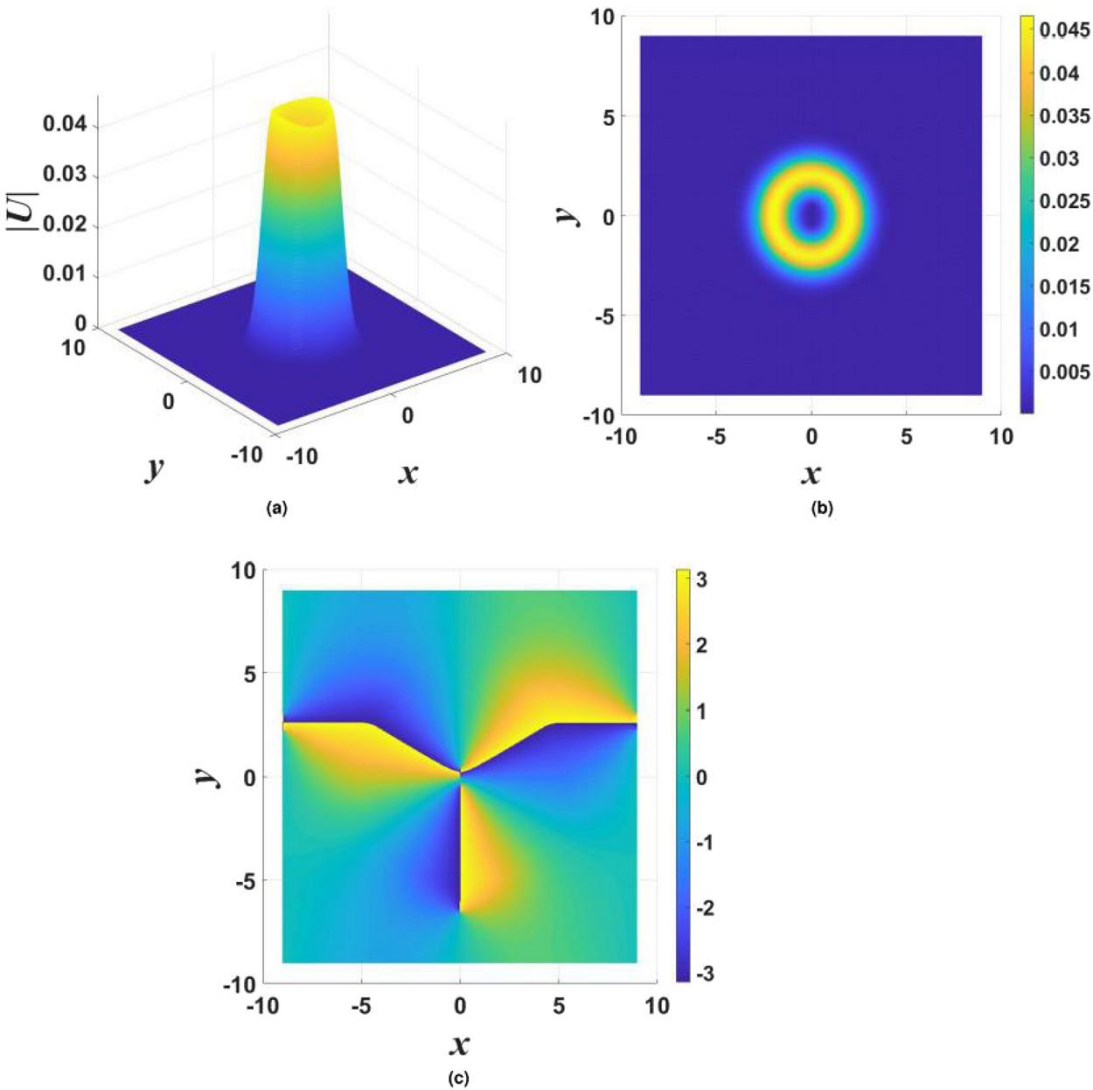
The same as in Fig. 13, but for stable vortex solitons with $m=3$ and parameters ${\gamma }_{0}=0.2$, $\beta =0.5$, $\sigma =0$, $k=-\,2.2$.

Starting from input Eq. (28), stable vortices can be constructed in the model with the gain-loss profile given by Eq. (4) only if it is subject to the spatial confinement (recall the same is reported above for zero-vorticity solitons). Examples of stable solitons with vorticities *m* = 1, 2 and 3 found in this model are shown in Figs 13, 14 and 15. Note that higher-order states with $m\ge 2$ are actually compound modes built of *m* unitary vortices, whose pivots do not merge into a single one, remaining separated, although with a small distance between them, as can be seen for $m=2$ in Fig. 14 (cf. a similar effect recently reported in ref.^84^). The separated pivots form arrays along axes *x* or *y*, the particular direction being randomly chosen by the initial conditions, as is clearly seen in Fig. 15. Nevertheless, the overall shapes of the unitary and higher-order vortices are nearly isotropic, due to the structure of the gain-loss term in Eq. (4) (cf. strongly anisotropic shapes of vortices in Figs 6, 8 and 9, supported by the imaginary potential (3)).

**Figure 16:**
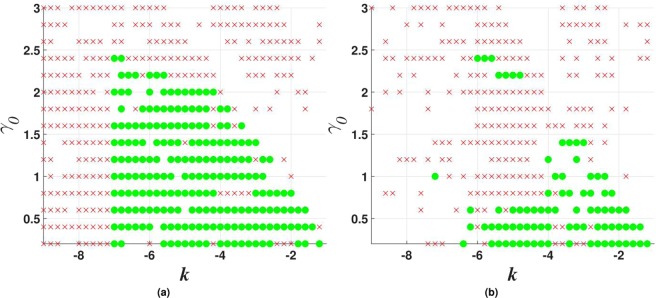
Stability charts for vortex solitons with $m=1$ in the model including imaginary potential (4), with $\beta =0.5$ and $\sigma =0$ in (**a**) or $\sigma =1$ in (**b**).

**Figure 17:**
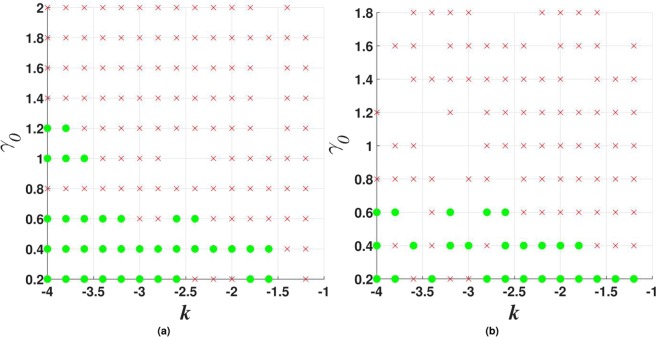
The same as in Fig. 16, but for vorticity $m=2$.

Stability charts obtained in this model for the solitons with embedded vorticities *m* = 1 and 2 are shown in Figs 16 and 17. Only few examples of stable vortices with *m* = 3 have been found in this case (for instance, the one shown in Fig. 15, as well as at $\sigma =0$, ${\gamma }_{0}=0.4$, $k=-\,1.2$).

**Figure 18:**
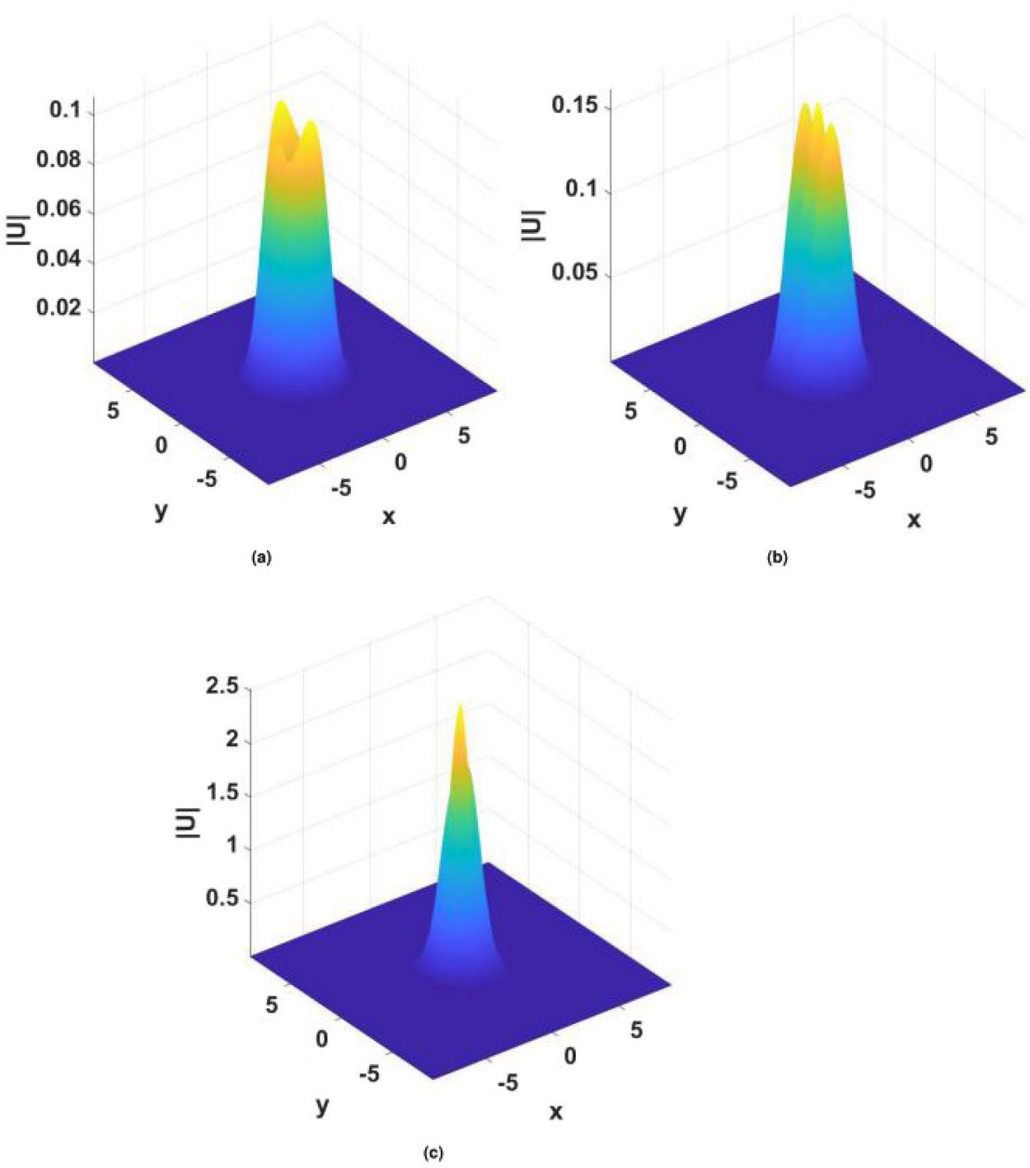
The blowup of an unstable vortex soliton with $m=2$ and ${\gamma }_{0}=1.2$, $\beta =0.5$, $\sigma =1$, $k=-\,2.4$, in the model with imaginary potential (4). Panels display the field at $z=60$ (**a**), $z=200$ (**b**) and $z=300$ (**c**). Note the difference in vertical scales between them.

Finally, a generic example of the evolution of an unstable vortex soliton is shown in Fig. 18. The strong difference between vertical scales in different panels of the figure clearly suggests that the instability leads to the blowup of the unstable mode, in the course of which the original vortex tends to fuse into a single peak. In fact, all unstable solitons considered in this work tend to develop the blowup in direct simulations.

## Discussion

The objective of this work is to elaborate 2D models with the spatially modulated self-defocusing nonlinearity and gain-loss distributions [imaginary potentials, *iW*(*x*, *y*)] which give rise to families of stable single-peak, multi-peak, and vortical solitons, including ones which may persist and remain stable (“unbreakable”) at arbitrarily large values of strengths *γ*_0_ of the imaginary potential. The unbreakability is possible in the case of the ${\mathscr{P}}{\mathscr{T}}$-symmetric imaginary potential, which is given by Eq. (3). An asset of the models, which can be implemented in bulk nonlinear optical waveguides with embedded gain and loss elements, is that they produce universal asymptotic solutions for solitons’ tails, along with full exact solutions for selected species of 2D fundamental and vortex solitons (the latter one is available in the elliptically deformed version of the model). In particular, in the limit of large *γ*_0_, the unbreakable family of fundamental solitons tends to shrink towards the exact solution. Generic families of zero-vorticity solitons, including single- and multi-peak ones and higher-order radial states of single-peak solitons, as well as families of self-trapped modes with embedded vorticity *m* = 1, 2, and 3, are constructed in the numerical form, and their stability is identified by means of the numerical computation of eigenvalues for small perturbations, and verified by direct simulations. In the case of the ${\mathscr{P}}{\mathscr{T}}$-symmetric imaginary potential (3) the solitons are stable in vast parameter regions, and feature a trend towards maintaining the unbreakable ${\mathscr{P}}{\mathscr{T}}$ symmetry. Under the action of the imaginary potential (4), families of stable fundamental and vortex solitons exist too, provided that the imaginary potential is subject to spatial confinement.

A relevant extension of the analysis may be to address the elliptically deformed model, which is considered in the present work in a brief form. A challenging problem is the possibility of the fractal structure of the stability patterns in the models’ parameter planes.

## Methods

### The Newton conjugate gradient method for the 2D robust *PT*-symmetry model

Solutions of the stationary Eq. (11) were constructed by means of the Newton conjugate gradient method, which is presented in detail in book^77^. In terms of this method, the stationary-solution operator **L**_0_ is defined by Eq. (11), while the respective linearization operator **L**_1_ is defined as

29 $${{\bf{L}}}_{1}=[\begin{array}{cc}A & B\\ C & D\end{array}],$$

with matrix elements

$$\begin{array}{rcl}A & = & -\,k+\frac{1}{2}{\nabla }^{2}-{\rm{\Sigma }}(r)\,([3{({\rm{Re}}U)}^{2}+{({\rm{Im}}U)}^{2}],\\ B & = & -\,2{\rm{\Sigma }}(r)\,{\rm{Re}}U\cdot {\rm{Im}}U+W(x,y),\\ C & = & -\,2{\rm{\Sigma }}(r)\,{\rm{Re}}U\cdot {\rm{Im}}U-W(x,y),\\ D & = & -\,k+\frac{1}{2}{\nabla }^{2}-{\rm{\Sigma }}(r)\,[3{({\rm{Im}}U)}^{2}+{({\rm{Re}}U)}^{2}],\end{array}$$

where the nonlinearity coefficient, ${\rm{\Sigma }}(r)$, and imaginary potential, *W*(*x*, *y*) are defined, respectively, by Eqs (3) or (4) and (9).

### Simulations of the evolution of the wave fields

Direct simulations of the evolution Eq. (8), written as

30 $$i\frac{\partial U}{\partial z}=-\,\frac{1}{2}\,(\frac{{\partial }^{2}U}{\partial {x}^{2}}+\frac{{\partial }^{2}U}{\partial {y}^{2}})+[k+{\rm{\Sigma }}(r)|U{|}^{2}+i\gamma (x,y)]\,U,$$

cf. Eq. (11), have been performed by means of the commonly known split-step method. Marching forward in *z* at each step was split in two parts, according to the following equations:

$$\begin{array}{rcl}{\bf{I}}:i\frac{\partial U}{\partial z} & = & [k+{\rm{\Sigma }}(r)|U{|}^{2}+i\gamma (x,y)]\,U,\\ {\bf{I}}{\bf{I}}:i\frac{\partial U}{\partial z} & = & -\,\frac{1}{2}\,(\frac{{\partial }^{2}U}{\partial {x}^{2}}+\frac{{\partial }^{2}U}{\partial {y}^{2}}).\end{array}$$

The solutions were numerically constructed in the 2D spatial domain, |*x*, *y*| ≤ 9, which was covered by a discrete grid of size ${N}_{x}\times {N}_{y}=512\times 512$. The direct simulations were carried out with step ${\rm{\Delta }}z={10}^{-5}$. This small step was selected to provide sufficient accuracy of the numerical solutions obtained in the presence of the “ exotic” nonlinearity-modulation and gain-loss profiles (9) and (3) or (4).

### The stability analysis

The stability of the stationary states against small perturbations were based, as usual, on the general expression for a perturbed solution,

31 $$u(x,y,z)={e}^{ikz}\,\{U\,(x,y)+\varepsilon \,[{e}^{{\rm{\Gamma }}z}v\,(x,y)+{e}^{{{\rm{\Gamma }}}^{\ast }z}{w}^{\ast }\,(x,y)]\},$$

where *ε* is an infinitesimal perturbation amplitude, with eigenmodes {*v*(*x*, *y*), *w*(*x*, *y*)} and (complex) eigenvalue Γ, which should be found from the numerical solution of the respective linearized equations,

32 $$\begin{array}{rcl}(\,-\,k+i{\rm{\Gamma }})\,v+\frac{1}{2}\,(\frac{{\partial }^{2}}{\partial {x}^{2}}+\frac{{\partial }^{2}}{\partial {y}^{2}})\,v-2{\rm{\Sigma }}(r)|U{|}^{2}v-{\rm{\Sigma }}{U}^{2}w & = & i\gamma \,(x,y)\,v,\\ (\,-\,k-i{\rm{\Gamma }})\,w+\frac{1}{2}\,(\frac{{\partial }^{2}}{\partial {x}^{2}}+\frac{{\partial }^{2}}{\partial {y}^{2}})\,w-2{\rm{\Sigma }}(r)|U{|}^{2}w-{\rm{\Sigma }}{U}^{2}v & = & -\,i\gamma \,(x,y)\,w,\end{array}$$

subject to zero boundary conditions at $|x,y|\to \infty$ (in fact, at borders of the solution domain). These equations were solved by means of the known spectral collocation method^77^.
